# Abnormal intrinsic functional hubs and connectivity in nurses with occupational burnout: a resting-state fMRI study

**DOI:** 10.3389/fpubh.2025.1595550

**Published:** 2025-06-16

**Authors:** Jian-Ping Liu, Si-Yu Gu, Chun-Mei Song, Hu-Cheng Yang, Yang Shi, Yu-Fang Gu, Shu-Fang Wang, Ying-Zhu Chen

**Affiliations:** ^1^School of Nursing and Public Health, Yangzhou University, Yangzhou, China; ^2^Department of Neurology, Yancheng Clinical Medical College of Yangzhou University, Yancheng Third People’s Hospital, Yancheng, China; ^3^Department of Radiology, Affiliated Hospital 6 of Nantong University, Yancheng Third People’s Hospital, Yancheng, China; ^4^Department of Disinfection Supply Center, Affiliated Hospital 6 of Nantong University, Yancheng Third People’s Hospital, Yancheng, China; ^5^Department of Radiology, Binhai Maternal and Child Health Hospital, Yancheng, China; ^6^Department of Neurology, Yangzhou Wutaishan Hospital of Jiangsu Province, Teaching Hospital of Yangzhou University, Yangzhou, China; ^7^Department of Geriatrics, Northern Jiangsu People’s Hospital Affiliated to Yangzhou University, Yangzhou, China

**Keywords:** burnout, resting-state functional MRI, degree centrality, functional connectivity, precuneus, medial orbitofrontal cortex

## Abstract

**Background:**

Occupational burnout is a significant problem among nurses, linked to negative outcomes. Understanding its neurobiological basis is crucial, yet remains limited.

**Methods:**

Resting-state functional magnetic resonance imaging (rs-fMRI) data were acquired from 40 female nurses with occupational burnout and 40 healthy controls. Degree centrality (DC) was calculated to identify functional hubs, and subsequent functional connectivity (FC) analysis was performed. Group differences in DC and FC were statistically compared. Their correlations with Maslach Burnout Inventory-Human Services Survey (MBI-HSS) scores were assessed, and a classification model was built using DC and FC features to distinguish between burnout and control groups.

**Results:**

The burnout group showed significantly decreased DC in bilateral precuneus and reduced FC between left precuneus and right medial orbitofrontal cortex (mOFC) compared to the healthy control group. These neuroimaging markers correlated with clinical burnout dimensions: precuneus DC negatively associated with emotional exhaustion and depersonalization, while precuneus-mOFC connectivity positively correlated with personal accomplishment. A linear discriminant analysis model combining DC and FC measures achieved 85% classification accuracy (sensitivity 80%, specificity 90%) in distinguishing burnout from controls.

**Conclusion:**

These findings identify the precuneus and its mOFC connectivity as key neural substrates of occupational burnout, suggesting disrupted integration between self-referential processing and reward/emotion regulation systems. Our results advance understanding of burnout’s neurobiological mechanisms and demonstrate the potential of neuroimaging markers for objective burnout assessment.

## 1 Introduction

Burnout is defined as a psychological state arising from chronic emotional or interpersonal stressors in the workplace (1). It manifests as an emotional and behavioral disorder caused by prolonged exposure to high occupational stress, particularly prevalent among nurses in the healthcare sector (2). Globally, approximately 30% of nurses experience occupational burnout (3), leading to increased turnover rates (4), economic losses (5), and compromised patient safety (6). The COVID-19 pandemic has exacerbated this crisis, intensifying psychological strain and accelerating burnout rates (7). Despite its severe societal and clinical implications, the neurobiological mechanisms underlying occupational burnout in nurse remain poorly understood, hindering early diagnosis and targeted interventions.

Over the past two decades, resting-state functional magnetic resonance imaging (rs-fMRI) has emerged as a powerful tool for investigating brain function alterations. By measuring blood oxygen level-dependent (BOLD) signals, rs-fMRI reveals spontaneous brain activity and the synchronization of neural networks in the absence of specific tasks (8, 9). Functional connectivity (FC), which assesses the temporal correlation of neural signals between brain regions, has been widely used to explore the intrinsic interactions within brain networks (10, 11). Previous studies have applied FC to investigate occupational burnout, identifying disrupted connectivity patterns (12–15); however, these efforts often rely on seed-based regions selected from prior literature, limiting their ability to capture the full scope of burnout-related neurobiological changes. Degree centrality (DC), a graph-based metric, offers an unbiased approach by quantifying the importance of brain regions as functional hubs within the connectome, reflecting their connectivity strength across the whole brain (16, 17). Combining DC and FC analyses may overcome this limitation by first identifying hub regions and then mapping their connectivity patterns, a strategy proven effective in neuropsychiatric disorders (17, 18).

This study aimed to investigate brain functional alterations in nurses with burnout using rs-fMRI. First, we employed DC analysis to identify abnormal functional hubs associated with burnout. Subsequently, these hubs served as regions of interest (ROIs) for whole-brain FC analysis to elucidate connectivity changes specific to burnout. Additionally, correlation analyses explored the relationship between these brain alterations and burnout severity, while machine learning techniques, integrating DC and FC features, was used to develop a classification model for distinguishing burnout cases from controls.

## 2 Methods

### 2.1 Subjects

This study recruited female nurses from Yancheng Clinical Medical College of Yangzhou University as subjects, with data collection conducted from September 2024 to December 2024. Inclusion criteria: the occupational burnout group: (1) female, aged 20–40 years; (2) right-handed; (3) according to the burnout norms of Chinese nurses (19), the critical value of burnout was determined as all three-dimensional scores exceeding critical values (emotional exhaustion (EE) ≥ 27 points, depersonalization (DP) ≥ 8 points, and personal accomplishment (PA) ≤ 24 points) and the healthy control group: (1) female, aged 20–40 years; (2) right-handed; (3) all three-dimension scores below critical values (EE < 27 points, DP < 8 points, PA > 24 points). Exclusion criteria: all subjects with any of the following conditions were excluded: (1) endocrine, neurological, or psychiatric disorders or other primary diseases; (2) pregnant or lactating women; (3) history of drug dependence, smoking, or alcohol consumption; (4) adverse reactions during scanning leading to termination of the experiment or contraindications to MRI scanning; (5) data collection failure during scanning or unclear images; (6) MRI images showing organic brain lesions; (7) other serious physical illnesses. Based on the inclusion and exclusion criteria, 80 subjects were ultimately selected, with 40 in the occupational burnout group and 40 in the heathy control group. The two groups were matched in terms of age and years of education. This study strictly adhered to the ethical principles of the Declaration of Helsinki and has received approval from the Ethics Committee of the Yancheng Clinical Medical College of Yangzhou University (2024–82) and obtained informed consent from all subjects involved.

Prior to MRI scanning, general information and clinical data were collected, including age, years of education, body mass index (BMI), Beck Anxiety Inventory (BAI) (20), Beck Depression Inventory-II (BDI-II) (21), and Maslach Burnout Inventory-Human Services Survey (MBI-HSS) scale. MBI-HSS scale assesses burnout across three dimensions in service industries: EE (9 items): emotional exhaustion from work; DP (5 items): depersonalized responses to care recipients; PA (8 items, reverse-scored): feelings of competence and achievement. Items are rated on a 7-point Likert scale (0: never to 6: daily) (22). Within our sample, the Cronbach’s *α* coefficients for the dimensions of EE, DP, and PA in the MBI-HSS were 0.970, 0.965, and 0.960, respectively.

### 2.2 MRI data acquisition

Rs-fMRI and structural 3D-T1-weighted images were acquired using a 3.0 T MRI scanner with a 24-channel head coil (Discovery 750w, GE, United States) at Yancheng Clinical Medical College of Yangzhou University. Parameters included: rs-fMRI: repetition time (TR)/ echo time (TE) = 3,000/35 ms, 128 volumes, field of view (FOV) = 24 cm × 24 cm, Slice thickness = 5.0 mm, and voxel size = 3.75 × 3.75 × 4 mm; structural 3D-T1: TR = 750 ms, TE = 2.8 ms, FOV = 24 cm × 24 cm, Slice thickness = 1.0 mm, number of slices = 152, flip angle = 15°, and voxel size = 0.5 × 0.5 × 1 mm.

### 2.3 Rs-fMRI preprocessing

Preprocessing was performed using the DPABI 8.2 software, including: (1) removal of the first 10 time points; (2) slice timing correction; (3) realignment for head motion; (4) exclusion of participants with maximum displacement > 3 mm or rotation > 3°; (5) spatial normalization to the standard Montreal Neurological Institute (MNI) space achieved through the Dartel alignment method; (6) linear regression to reduce errors; (7) regression of nuisance covariates; and (8) band-pass filtering (0.01–0.1 Hz). All participants included in the final analysis met these head motion criteria.

### 2.4 Total intracranial volume (TIV) extraction

Brain structural 3D-T1-weighted images were preprocessed using SPM12 and CAT12, including bias field correction, skull stripping, alignment to MNI template, and segmentation into gray matter, white matter, and cerebrospinal fluid. TIV was extracted for all participants.

### 2.5 DC analysis

DC was calculated using DPABI 8.2, computing the DC value for each voxel in the brain. Pearson correlation coefficients were used to estimate functional connectivity between all pairs of gray matter voxels. A threshold of r > 0.25 was used to derive the adjacency matrix, followed by conversion of individual voxelwise DC values into a z-score map. Subsequently, the DC maps obtained were smoothed spatially using a 6-mm full width at half-maximum (FWHM) Gaussian kernel.

### 2.6 FC analysis

Using the AAL 90 template, brain regions with significant DC differences between the burnout and control groups were selected as ROIs for whole-brain FC analysis. The average time series for each ROI was calculated, and Fisher’s z-transformation was applied to obtain normally distributed z-score maps.

### 2.7 Statistical analysis

SPSS 27.0 software was used for statistical analysis. First, normality tests were conducted for age, years of education, BMI, TIV, BAI, BDI-II, and MBI-HSS. For normally distributed measurement data, independent sample *t*-tests were used, while non-parametric tests were applied for skewed distribution data. A *p*-value < 0.05 was considered to indicate significant between-group differences.

Two-sample *t*-tests were conducted to compare differences in FC and DC between the burnout group and the control group, with age, years of education, TIV, BAI, and BDI-II as covariates using SPM12 in MATLAB (R2020b). Multiple comparison corrections used cluster-level False Discovery Rate (FDR), with voxel-level *p* < 0.001 and cluster-level *p* < 0.05. DC and FC values from ROIs (showing significant group differences) were extracted for subsequent correlation and machine learning analyses.

### 2.8 Correlation analysis

SPSS 27.0 software was used to conduct Spearman correlation analysis between the extracted brain DC and FC signal values with significant between-group differences and burnout-related scales (EE, DP, PA). A *p*-value < 0.05 was considered to indicate significant correlation.

### 2.9 Classification model construction

This study used DC values and FC values from brain regions with significant differences obtained through two-sample *t*-tests and cluster-level FDR correction, as well as combined DC and FC values, as input features for Linear discriminant analysis (LDA) to construct classification models for burnout and healthy populations. The predictive performance of the models was evaluated using receiver operating characteristic (ROC) curves, calculating the area under curve (AUC), sensitivity, specificity, and accuracy, and comparing the performance of these three models in the validation set. Leave-one-out cross-validation (LOOCV) was used to validate the predictive ability and stability of the classification models. Subsequently, permutation tests were conducted to evaluate the significance of classification accuracy and AUC. During the permutation tests, group labels were randomly shuffled 5,000 times, and classification was performed on the newly generated datasets, calculating the classification accuracy and AUC each time. The *p*-value for classification accuracy and AUC was the number of times the classification accuracy or AUC in the 5,000 random cases exceeded the true value, divided by 5,000. Results were considered significant when the *p*-values for both accuracy and AUC were less than 0.05.

## 3 Results

### 3.1 Demographic and clinical characteristics

The study included 40 participants in occupational burnout group and healthy control group. Demographic and clinical characteristics of both groups are summarized in Table 1. No statistically significant differences were observed between the occupational burnout and healthy control groups in age (median [interquartile range, IQR]: 33 [27–37] vs. 33.5 [28–36] years; *p* = 0.973), years of education (median [IQR]: 16 [16–16] vs. 16 [16–16]; *p* = 0.724), or body mass index (BMI; median [IQR]: 21.89 [19.64–23.81] vs. 20.81 [19.55–23.63]; *p* = 0.544). The occupational group exhibited markedly higher anxiety levels on the BAI (median [IQR]: 27 [23.25–33] vs. 24 [22–26]; *p* = 0.009) and greater depressive symptoms on the BDI-II (median [IQR]: 9.5 [5.5–13.75] vs. 4 [0–9.5]; *p* = 0.002). Scores on MBI-HSS subscales further distinguished the groups. The occupational group reported significantly higher EE (median [IQR]: 36.0 [30.5–40.00] vs. 13.5 [9–17]; *p* < 0.001) and DP (median [IQR]: 13.0 [10.0–16.75] vs. 3 [0–5]; *p* < 0.001), alongside significantly lower PA (median [IQR]: 20.0 [17.25–22.0] vs. 38.5 [30–43.75]; *p* < 0.001) compared to the healthy control group.

**Table 1:** Demographic information and clinical data.

Characteristics	Nurses with burnout	HCs	*p*
N	40	40	-
Age (years)	33 (27, 37)	33.5 (28, 36)	0.973^a^
Education (years)	16 (16,16)	16 (16,16)	0.724^a^
TIV (mm^3^)	1417.49 (1341.05, 1512.72)	1412.13 (1376.92, 1518.53)	0.679^a^
SBP (mmHg)	118 (103.50, 122.50)	120 (106.25, 120)	0.675^a^
DBP (mmHg)	70 (62.50, 76)	70 (65, 80)	0.317^a^
BMI	21.89 (19.64, 23.81)	20.81 (19.55, 23.63)	0.544^a^
EE	36.0 (30.5, 40)	13.5 (9, 17)	*<0.001* ^a^
DP	13.0 (10.0, 16.75)	3 (0, 5)	*<0.001* ^a^
PA	20.0 (17.25, 22.0)	38.5 (30, 43.75)	*<0.001* ^a^
BAI	27 (23.25, 33)	24 (22, 26)	*0.009* ^a^
BDI-II	9.5 (5.5, 13.75)	4 (0, 9.5)	*0.002* ^a^

Data are represented as median (quartiles). Italic represents a significant difference between two groups of people. HCs, healthy controls; TIV, intracranial total volume; SBP, systolic blood pressure; DBP, diastolic blood pressure; BMI, body mass index; EE, emotional exhaustion; DP, depersonalization; PA, reduced personal accomplishment; BAI, beck anxiety inventory; BDI-II, beck depression inventory-II.

a The p values were acquired through Mann–Whitney U test.

### 3.2 Group differences in DC

**Figure 1:**
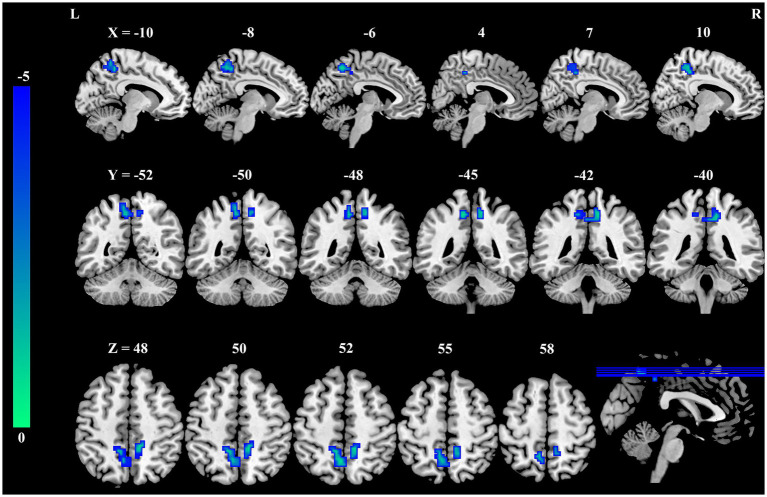
Group differences in DC between female nurses with occupational burnout and HCs. Compared to HCs, nurses with burnout showed a significant decrease in DC in the bilateral PCUN. DC, degree centrality; HCs, healthy controls; PCUN, precuneus.

As shown in Table 2 and Figure 1, significant differences in DC were observed between the occupational burnout group and the healthy control group. After applying cluster-level FDR correction (voxel threshold: *p* < 0.001 and cluster threshold: *p* < 0.05), the occupational burnout group exhibited significantly reduced DC values in the left precuneus (*t* = −4.37, *p* < 0.001) and the right precuneus (*t* = −4.55, *p* < 0.001) compared to the healthy control group.

**Table 2:** Group differences in DC between nurses with occupational burnout and HCs.

Cluster	Regions (AAL)	Cluster size	MNI coordinate (mm)			*t*
			x	y	z	
Cluster 1	PCUN. R	40	12	−42	45	−4.55
Cluster 2	PCUN. L	51	−6	−57	48	−4.37

Compared to HCs, nurses with burnout showed a significant decrease in DC in the bilateral precuneus. DC, degree centrality; HCs, healthy controls; AAL, anatomical automatic labeling; MNI, Montreal Neurological Institute; PCUN, precuneus; L, left; R, right.

### 3.3 Group differences in FC

**Figure 2:**
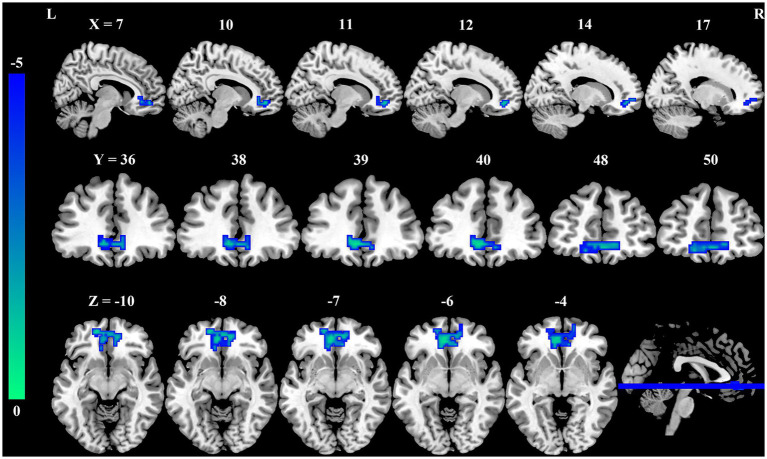
Group differences in FC between female nurses with occupational burnout and HCs. Compared to HCs, FC between the PCUN. L and mOFC. R is significantly reduced in nurses with burnout. FC, functional connectivity; HCs, healthy controls; PCUN, precuneus; mOFC, medial orbitofrontal cortex; L, left; R, right.

Seed-based whole-brain FC analysis was conducted using the DC-differentiated brain regions as seeds. As summarized in Table 3 and illustrated in Figure 2, the occupational burnout exhibited significantly reduced FC values between the left precuneus and the right medial orbitofrontal cortex (mOFC, *t* = −4.57, *p* < 0.001) compared to the healthy control group.

**Table 3:** Group differences in FC between nurses with occupational burnout and HCs.

Seed region	Regions (AAL)	Cluster size	MNI coordinate (mm)			*t*
			x	y	z	
PCUN. L	mOFC. R	111	12	45	−12	−4.57

Compared to HCs, FC between the PCUN. L and mOFC. R is significantly reduced in nurses with burnout. FC, functional connectivity; HCs, healthy controls; PCUN, precuneus; AAL, anatomical automatic labeling; MNI, Montreal Neurological Institute; mOFC, medial orbitofrontal cortex; L, left; R, right.

### 3.4 Correlation analysis

**Figure 3:**
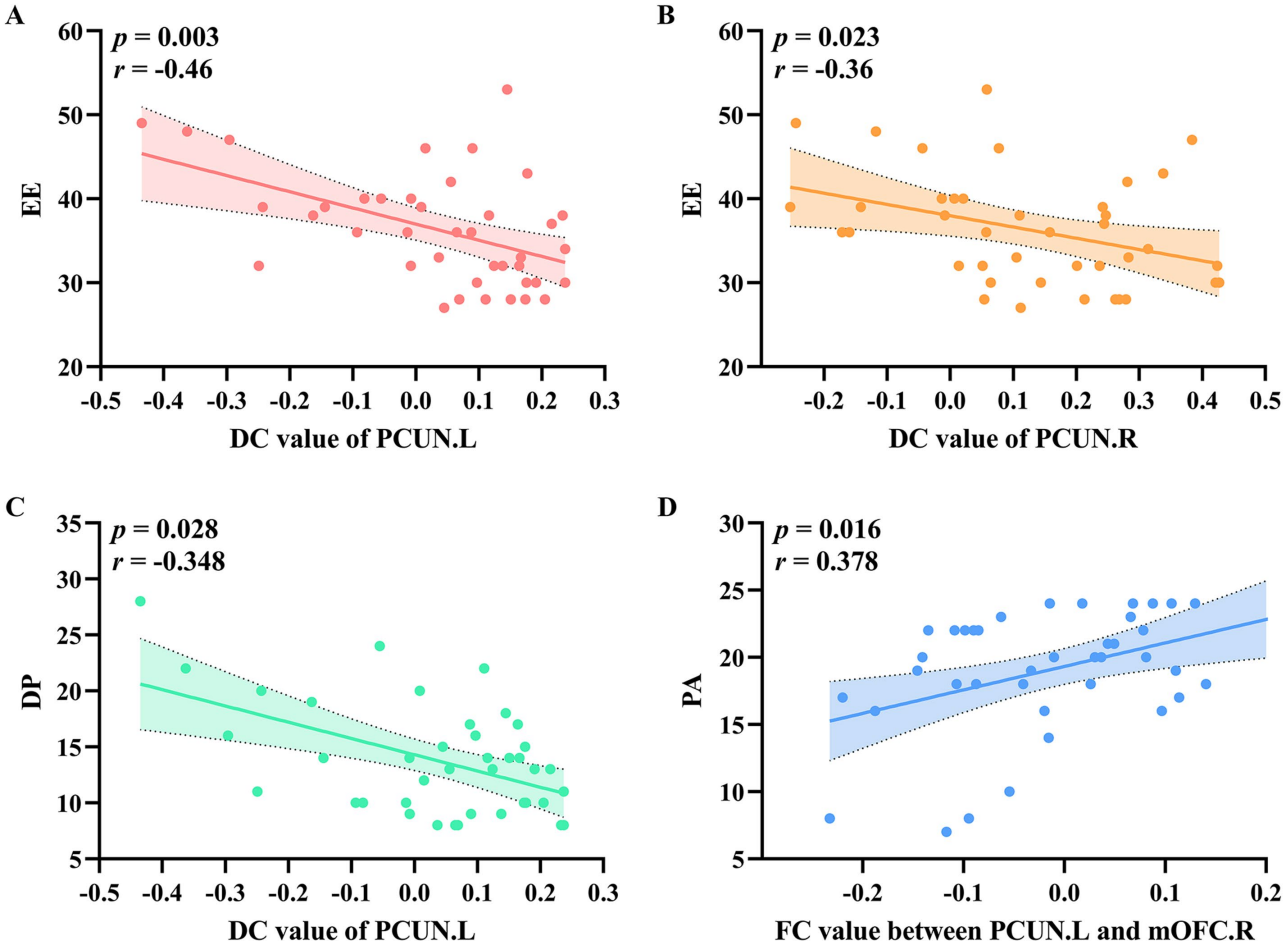
The relationship between DC values and FC values with clinical variables in the occupational burnout group. **(A)** EE was negatively correlated with DC values in the left precuneus. **(B)** EE was negatively correlated with DC values in the right precuneus. **(C)** DP was negatively correlated with DC values in the left precuneus. **(D)** PA was positively correlated with FC values between the left precuneus and the right orbital part of the superior frontal gyrus. DC, degree centrality; FC, functional connectivity; EE, emotional exhaustion; DP, depersonalization; PA, reduced personal accomplishment.

As illustrated in Figures 3A,B, EE was negatively correlated with DC values in the left precuneus (*r* = −0.46, *p* = 0.003) and right precuneus (*r* = −0.36, *p* = 0.023). Additionally, DP was negatively correlated with DC values in the left precuneus (Figure 3C) (*r* = −0.348, *p* = 0.028). As shown in Figure 3D, PA was positively correlated with FC values between the left precuneus and the right mOFC (*r* = 0.378, *p* = 0.016).

### 3.5 Classification model analysis

**Figure 4:**
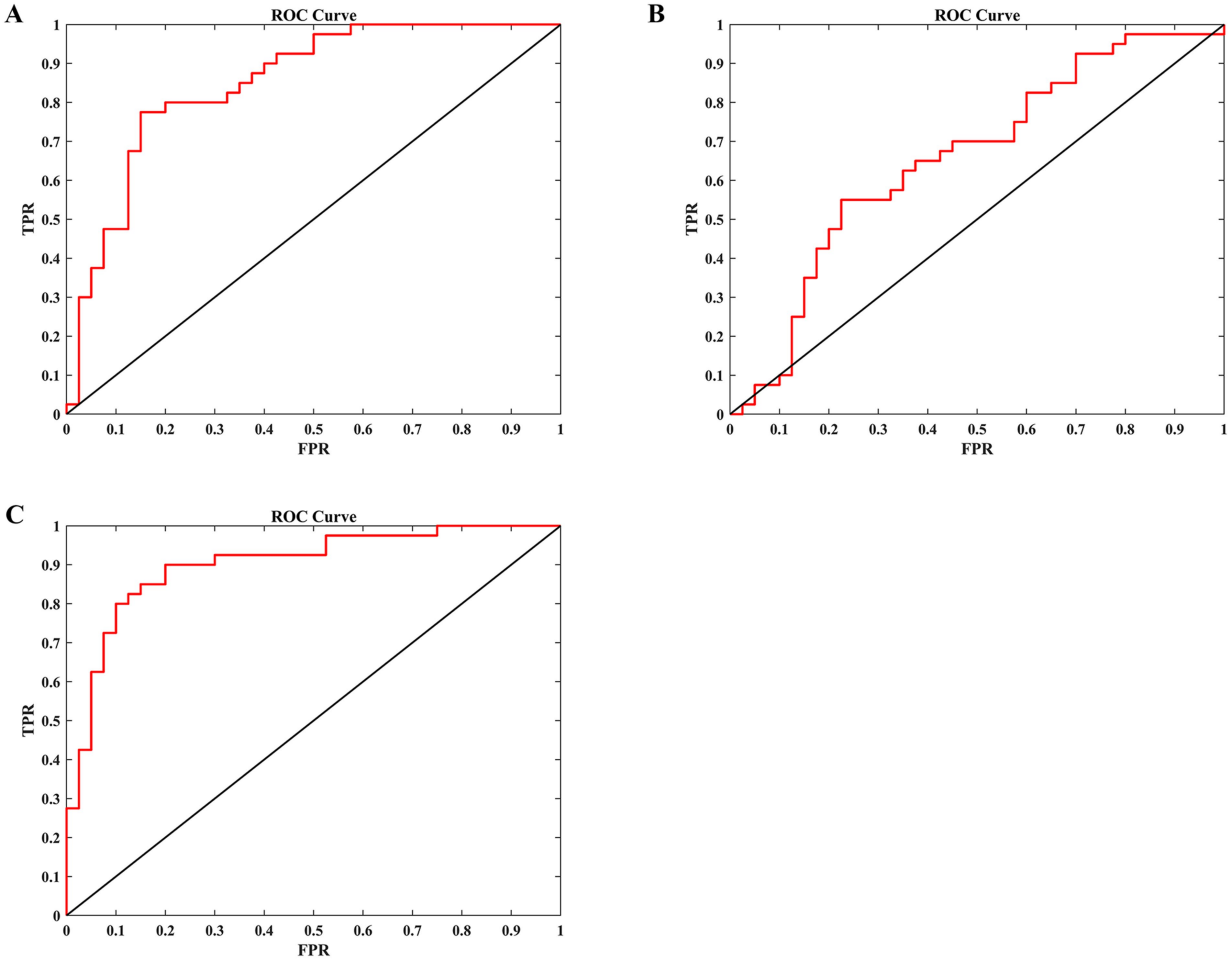
The ability of an LDA model to differentiate between nurses with burnout and HCs based on different input features. **(A)** Use the DC values of the bilateral PCUN as input features. **(B)** Use the FC values between the PCUN. L and the mOFC. R as input features. **(C)** Use both the DC values of the bilateral precuneus and the FC values between the PCUN. L and the mOFC. R as input features. LDA, linear discriminant analysis; HCs, healthy controls; DC, degree centrality; FC, functional connectivity; PCUN, precuneus; mOFC, medial orbitofrontal cortex; L, left; R, right; ROC, receiver operating characteristic; TPR, true positive rate; FPR, false positive rate.

To evaluate the classification performance, we employed LOOCV LDA model to build models distinguishing occupational burnout individuals from healthy controls. Input features for these models were DC and FC values extracted from brain regions exhibiting significant group differences. As shown in Table 4 and Figure 4, the LDA model incorporating both DC and FC values achieved the highest performance (sensitivity = 0.800, specificity = 0.900, accuracy = 0.850, and AUC = 0.902), significantly outperforming models using only DC values (sensitivity = 0.800, specificity = 0.675, accuracy = 0.738, and AUC = 0.851) or only FC values (sensitivity = 0.575, specificity = 0.675, accuracy = 0.625, and AUC = 0.657).

**Table 4:** The ability of an LDA model to differentiate between nurses with occupational burnout and HCs based on different input features.

Model	Sensitivity	Specificity	Accuracy	AUC
Model 1	0.800 (*p* < 0.001)	0.675 (*p* = 0.036)	0.738 (*p* < 0.001)	0.851 (*p* < 0.001)
Model 2	0.575 (*p* = 0.348)	0.675 (*p* = 0.015)	0.625 (*p* = 0.038)	0.657 (*p* = 0.008)
Model 3	0.800 (*p* < 0.001)	0.900 (*p* < 0.001)	0.850 (*p* < 0.001)	0.902 (*p* < 0.001)

The LDA model, which included both DC and FC values, outperformed models that used only DC or FC values individually. Model 1: Use the DC values of the bilateral PCUN as input features. Model 2: Use the FC values between the PCUN. L and the mOFC. R as input features. Model 3: Use both the DC values of the bilateral precuneus and the FC values between the PCUN. L and the mOFC. R as input features. LDA, linear discriminant analysis; HCs, healthy controls; AUC, area under curve; DC, degree centrality; FC, functional connectivity; PCUN, precuneus; mOFC, medial orbitofrontal cortex; L, left; R, right.

## 4 Discussion

This study investigated the neural correlates of occupational burnout in female nurses by examining alterations in DC and corresponding FC using rs-fMRI. Our findings revealed significant reductions of DC within the bilateral precuneus, and decreased FC between the left precuneus and the right mOFC in the occupational burnout group compared to healthy control group. Furthermore, we observed significant correlations between these neuroimaging markers and clinical burnout dimensions and demonstrated the potential of combining DC and FC measures to effectively classify individuals with occupational burnout.

The decreased DC in the precuneus in the occupational burnout group was a key finding observed in our study. The precuneus is a central hub within the default mode network (DMN), widely recognized for its involvement in a range of higher-order cognitive functions, including self-referential processing, introspection, episodic memory recall, and maintaining awareness (23–25). Reduced DC in this region suggests a disruption in its functional integration and potentially altered processing of internal states in individuals experiencing burnout. This aligns with the core features of burnout, which include emotional exhaustion and a sense of detachment from work, both of which may be linked to altered self-awareness and introspective abilities (26–28). Previous studies have also implicated the precuneus in stress-related conditions and emotional regulation (29–31), further supporting our findings. The negative correlations between precuneus DC and both emotional exhaustion and depersonalization dimensions of the MBI-HSS further reinforced this interpretation, suggesting that lower precuneus centrality is associated with higher levels of burnout severity in these domains.

Beyond the hub properties of the precuneus, the observed reduction in FC between the left precuneus and the right mOFC offers deeper insights into the neurobiological pathways affected by burnout. The mOFC is firmly established as a pivotal area for processing reward, regulating emotions, and guiding decision-making processes (32–35). The precuneus and mOFC are anatomically and functionally interconnected regions (23, 32), and their coordinated activity is likely essential for integrating self-referential information with emotional and regulatory processes. The decreased FC between these regions in burnout may indicate a disruption in this crucial communication pathway, potentially explaining why burnout patients often experience symptoms such as emotional exhaustion, reduced sense of work value, cognitive function decline, and decreased responsiveness to work-related rewards (26–28, 36, 37). Interestingly, we found a positive relationship between this precuneus-mOFC connectivity and personal accomplishment, a key facet of burnout. This suggests that stronger functional integration within this neural circuit may be indicative of greater professional self-efficacy and enhanced resilience against burnout. It is conceivable that robust connectivity here facilitates a more effective integration of self-perception with the emotional and motivational systems pertinent to work, thereby providing a buffer against the development of burnout (26).

Moving beyond the neurobiological findings, the performance of our classification model points towards potential clinical utility. The superior performance of the LDA model when incorporating both DC and FC values underscores the complementary nature of these measures in capturing the neurobiological underpinnings of burnout. The high accuracy, sensitivity, and specificity achieved by the combined model, particularly with LOOCV validation, suggest that these rs-fMRI-derived metrics hold promise as objective biomarkers. Such biomarkers could be invaluable in aiding the identification and diagnosis of occupational burnout, especially considering the inherent subjectivity of current burnout assessment tools and the recognized need for more objective measures within both clinical and occupational health contexts.

While the present study offers valuable perspectives on the neural mechanisms associated with occupational burnout, it is important to acknowledge certain limitations. Firstly, this study focuses solely on female nurses aged 20–40, recruited from a single center, potentially limiting the generalizability of our findings. Future multicenter studies should incorporate more diverse samples to validate these results in a broader population and varied institutional settings. Secondly, the cross-sectional nature of our study design prevents us from establishing definitive causal relationships between the observed brain functional changes and the development of burnout. Longitudinal investigations are essential to clarify whether these neuroimaging alterations precede the onset of burnout or emerge as a consequence of prolonged occupational stress. Thirdly, while DC and FC provided a valuable initial window into neural function, future studies could benefit from exploring a wider array of neuroimaging measures. Examining cerebral blood flow, as well as structural and functional network organization, could offer a more comprehensive and nuanced understanding of the neural underpinnings of burnout. Fourthly, a significant limitation of our study is the lack of control over menstrual cycle phases or the use of hormonal contraceptives, as our research focused solely on female nurses. Given that hormonal fluctuations can impact emotional regulation and rs-fMRI signals, future studies should systematically consider these factors to gain a more comprehensive understanding of their potential effects on brain function in the context of burnout. Fifthly, while LOOCV was employed for model validation and is suitable for the current sample size, its performance on this dataset does not guarantee generalizability to entirely new, independent populations. Future research should rigorously test these neuroimaging-based classifiers on larger, external datasets, ideally using independent held-out test sets, to establish their robustness and potential for broader clinical application. Finally, while our sample size was adequate for detecting statistically significant group differences, replication of these findings in larger cohorts would further strengthen the robustness and generalizability of our conclusions.

## 5 Conclusion

In summary, this study identifies reduced DC in the precuneus and its decreased FC with the mOFC as key neural substrates of occupational burnout, indicating impaired integration between self-referential processing and reward/emotion regulation systems. Furthermore, the significant correlations observed with clinical burnout scales, coupled with the robust diagnostic accuracy of our integrated DC-FC model, underscore the considerable potential of neuroimaging biomarkers for the objective assessment of burnout. These findings contribute to a better understanding of the pathophysiological mechanisms underlying burnout and may pave the way for the development of targeted neuromodulation therapies.

## Data Availability

The original contributions presented in the study are included in the article/supplementary material, further inquiries can be directed to the corresponding author.

## Glossary

AAL: anatomical automatic labeling
AUC: area under curve
BAI: Beck Anxiety Inventory
BDI**-II**: Beck Depression Inventory-II
BMI: body mass index
BOLD: blood oxygen level-dependent
DC: degree centrality
DMN: default mode network
DP: depersonalization
EE: emotional exhaustion
FC: functional connectivity
FDR: False Discovery Rate
FOV: field of view
FPR: false positive rate
FWHM: full width at half-maximum
HCs: healthy controls
IQR: interquartile range
L: left
LDA: linear discriminant analysis
LOOCV: leave-one-out cross-validation
MBI**-HSS**: Maslach Burnout Inventory-Human Services Survey
MNI: Montreal Neurological Institute
mOFC: medial orbitofrontal cortex
PA: personal accomplishment
PCUN: precuneus
R: right
ROC: Receiver Operating Characteristic
ROIs: regions of interest
rs**-fMRI**: resting-state functional magnetic resonance imaging
TE: echo time
TIV: total intracranial volume
TPR: true positive rate
TR: repetition time
3D**-T1**: structural 3D-T1-weighted
